# Proteomics of *Nasonia vitripennis* and the effects of native *Wolbachia* infection on *N. vitripennis*

**DOI:** 10.7717/peerj.4905

**Published:** 2018-05-28

**Authors:** Jie Li, Ningxin Wang, Yong Liu, Shiqi Qiu

**Affiliations:** Department of Entomology, College of Plant Protection, Shandong Agricultural University; Shandong Provincial Key Laboratory for Biology of Vegetable Diseases and Insect Pests, Shandong Agricultural University, Taian, Shandong, China

**Keywords:** *Wolbachia*, Immunity, Proteomics, iTRAQ, *Nasonia vitripennis*

## Abstract

**Background:**

*Nasonia vitripennis*, a parasitic wasp, is a good model organism to study developmental and evolutionary genetics and to evaluate the interactions between insect hosts and their symbionts. *Wolbachia* may be the most prevalent endosymbiont among insect species due to their special ability to improve the fitness of the infected hosts. Transinfection of bacteria or fungi could substantially alter the expression of host immune system components. However, few studies have focused on the effects of native *Wolbachia* infection. Accordingly, in this study, we evaluated the proteomics of *N. vitripennis* following *Wolbachia* infection.

**Methods:**

We studied the proteomics of *N. vitripennis* following native *Wolbachia* infection and in antibiotic-treated *Wolbachia*-free samples using isobaric tags for relative and absolute quantification-liquid chromatography tandem mass spectrometry, accompanying with some ecological experiments.

**Results:**

In total, 3,096 proteins were found to be associated with a wide range of biological processes, molecular functions, and cellular components. Interestingly, there were few significant changes in immune or reproductive proteins between samples with and without *Wolbachia* infection. Differentially expressed proteins were involved in the binding process, catalytic activity, and the metabolic process, as confirmed by quantitative reverse transcription polymerase chain reaction.

**Discussion:**

Invasion of any pathogen or bacterium within a short time can cause an immunoreaction in the host. Our results implied that during the long process of coexistence, the immune system of the host was not as sensitive as when the symbiont initially infected the host, implying that the organisms had gradually adjusted to cohabitation.

## Introduction

*Nasonia* is the second genus of Hymenoptera to have been subjected to whole-genome sequencing, assembly, and annotation, after *Apis mellifera* (Werren et al., 2010). *Nasonia vitripennis* (Hymenoptera: Pteromalidae), a parasitic wasp, lay their eggs into the pupa of many different flies; the eggs then develop into adults in pupa, and during this process, the wasps act as natural enemies to flies (Dong et al., 2009). *N. vitripennis* is becoming a model organism for developmental and evolutionary genetics (Shuker, Lynch & Peire Morais, 2003; Werren & Loehlin, 2009b) and for the study of interactions between insect hosts and symbionts owing to its overall ease of laboratory use, short generation time (roughly two weeks), tolerance for inbreeding, and straightforward rearing (Li et al., 2017).

The close relationship between *N. vitripennis* and the maternally inherited endosymbiont *Wolbachia* has been extensively studied. *Wolbachia*, an intracellular gram-negative bacterium, naturally infects up to 40% of arthropod species (Werren, Baldo & Clark, 2008; Zug & Hammerstein, 2012). Because *Wolbachia* is transmitted through the female germline, it has evolved a number of reproductive strategies to favor the *Wolbachia*-infected females (Sullivan, 2016), such as cytoplasmic incompatibility (CI), male-killing, induction of parthenogenesis, feminization, and speciation (Werren, 1997; Werren, Baldo & Clark, 2008). Among these manipulations, the most common effect is CI (Tram & Sullivan, 2002), whereby infected females mated with infected or uninfected males produce viable embryos, while uninfected females mated with infected males produce inviable embryos (Sullivan, 2016). The reproductive effect of *Wolbachia* on *N. vitripennis* is just CI (Breeuwer & Werren, 1993) and can be influenced by temperature, bacterial density, and the bacteriophage WO (Bordenstein & Bordenstein, 2011; Breeuwer & Werren, 1993). Although there are tight associations between *Wolbachia* and insect hosts, they do not evolve simultaneously due to *Wolbachia* horizontal transmission, even between phylogenetically distant species (Wang et al., 2016; Zug, Koehncke & Hammerstein, 2012).

In addition to reproductive modifications, the global spread of *Wolbachia* has also been attributed to increased host fecundity and protection against pathogens (Hedges et al., 2008; Teixeira, Ferreira & Ashburner, 2008; Zug & Hammerstein, 2015a). *Wolbachia* confers resistance to various pathogens, e.g., by priming the innate immune system in *Aedes aegypti* (Bian et al., 2010; Moreira et al., 2009). Insects rely on the innate immune system to mount defense responses against pathogenic invasions (Rodrigues et al., 2010). The insect innate immune system consists of humoral immune and cellular immune responses (Govind, 2008; Rolff & Siva-Jothy, 2003). Humoral immune responses involve immune-related molecules, such as antimicrobial peptides, lysozyme, or phenoloxidase (Urbanski et al., 2014), whereas the main cellular immune responses involve pathogen phagocytosis, nodulation, and encapsulation (Lanz-Mendoza et al., 1996).

Studies have shown that invasion of any pathogen or bacterium within a short time can cause an immunoreaction in the host. The genome-wide analysis of the interaction between *Wolbachia* and its *Drosophila* host showed involvement of an antimicrobial humoral response and negative regulation of cell proliferation in the host (Wong et al., 2011). In silkworms, after transinfection with *Bacillus subtilis* (a gram-negative bacteria) for 24 h, 2,436 genes showed more than two-fold changes in expression (Huang et al., 2009), and the systemic immune response was triggered via the Toll-like receptor pathway, resulting in changes in the expression of antimicrobial peptide genes, such as *Attacin*, *Lebocin*, *Enbocin*, *Gloverin*, and *Moricin* (Huang et al., 2009). Such transinfections usually induce a higher bacterial density and cause other alterations to host physiology (McMeniman et al., 2009; Pan et al., 2012).

Compared with transinfection, native or natural infection by *Wolbachia* has not been extensively studied, except for a few studies in mosquitoes (Hughes et al., 2011b; Joubert & O’Neill, 2017; McMeniman et al., 2009). Stable infection of *Wolbachia* induced the up- or downregulation of 257 transcripts, with no changes in Toll and immune deficiency (IMD) pathway genes in *Aedes fluviatilis* (Caragata et al., 2017). Hughes et al. (2011a) found that the immune response in *Anopheles* after somatic infection is dynamic, as it was first induced and then suppressed as the infection progressed. However, the effects on immune-related genes in the host following long-term coevolution with *Wolbachia* are still unclear.

Accordingly, in this study, we used native *Wolbachia*-infected and antibiotic-treated *Wolbachia*-free *N. vitripennis* to explore how *Wolbachia* affects host biology at the protein level by combining isobaric tags for relative and absolute quantification-liquid chromatography tandem mass spectrometry (iTRAQ-LC-MS/MS) and some ecological experiments. Overall, 3,096 *N. vitripennis* proteins were found to be associated with various biological processes (BPs), molecular functions (MFs), and cellular components (CCs). However, few changes were observed in immune or reproductive proteins, suggesting that during coevolution, the impacts of long-term infection of *Wolbachia* gradually declined.

## Materials and Methods

### *Nasonia* materials

Two *Nasonia* lines were used in our experiments, native *Wolbachia-*infected *N. vitripennis* and *Wolbachia*-free *N. vitripennis*; both lines had been grown in our laboratory since 2011. The *Wolbachia*-infected line was naturally infected with single Supergroup A *Wolbachia* strain, as confirmed by *Wolbachia* surface protein (WSP). The *Wolbachia*-free *N. vitripennis* line was treated with rifampin to remove native *Wolbachia* at the beginning, and repeated polymerase chain reaction (PCR) with primers targeting the WSP gene was used to confirm the effects of removal. *Wolbachia-*free organisms used in our study were reared after 30 generations to reduce the effects of antibiotics. Two lines were maintained using standard insectary conditions at 28 °C for light and 25 °C for dark (L:D = 16:8 h) with 60% ± 10% relative humidity (Werren & Loehlin, 2009c). In our experiment, *N. vitripennis* parasitised *Wolbachia*-free *Sarcophaga marshalli* pupa, where the eggs, larvae, and pupae of the wasp were developed (Werren & Loehlin, 2009c), and the adults were reared with 10% honey water.

### Ecological experiment

At 10–13 days after *N. vitripennis* oviposited into *Sarcophaga marshalli* pupae, the pupae were dissected to obtain virgin adults. The male and female pupa were identified individually and collected separately once upon eclosion. Four crossing types based on different *Wolbachia* infection statuses were carried out (♀ W+ × ♂ W+, ♀ W+ × ♂ W−, ♀ W− × ♂ W−, ♀ W− × ♂ W+). A virgin female and a virgin male adult were placed in a 7 mL tube, together with one fresh *Sarcophaga marshalli* pupa for oviposition. The offspring of the crossings were collected and counted, and the percent females (the proportion of females of the total number of individuals) was calculated. The offspring number and percent females based on the number of fly pupae per day were calculated for nine consecutive generations. Moreover, the effects of *Wolbachia* on longevity of *N. vitripennis* were studied. Approximately 300 samples were used for life testing. Each experiment was repeated with six groups.

### Protein preparation

*Wolbachia-*infected and *Wolbachia-*free line were prepared for iTRAQ separately, each line contains three replicates. The female adults were collected after eclosion two to three days, and all adults of a sample were from the same generation. Approximately 200 adult wasps per sample were homogenized, and total proteins were extracted using the phenol extraction procedure. The protein content in the supernatant was quantified using a BCA Protein Assay Kit (Thermo Scientific, Milford, MA, USA).

### Proteome analysis by iTRAQ

The proteome was quantified using iTRAQ-LC-MS/MS. Protein abundances that changed more than 1.2-fold were regarded as significantly differentially expressed. The extracted proteins were digested, and peptides were quantified as described by Wisniewski et al. (2009). For peptide labeling, the peptide mixture from each group was labeled with iTRAQ Reagent-8plex multiplex kit (AB SCIEX, Foster City, CA, USA) according to the manufacturer’s instructions. *Wolbachia-*free and *Wolbachia-*infected samples were labeled with iTRAQ reagents 113, 115, 117 and 114, 116, 118, respectively. The labeled peptide mixtures were pooled for strong cation-exchange chromatography. Samples were separated by nanoLC-MS/MS (EASY-nLC1000). The mass spectrometry data were analyzed using a Q-Exactive mass spectrometer (Thermo Finnigan, Waltham, MA, USA) in the positive ion mode with a selected mass range of *m/z* 300–1,800.

### Quantitative real-time reverse transcription PCR

Total RNA was isolated using *TransZol* UP (TRANSGEN BIOTECH, Beijing, China) from *Wolbachia*-infected and *Wolbachia*-free *N. vitripennis* adults (50 individuals each). The first-strand cDNA was reverse-transcribed from 2 μg total RNA using *TransScript* One-Step gDNA Removal and cDNA Synthesis SuperMix (TRANSGEN BIOTECH, Beijing, China) at 42 °C for 15 min according to the manufacturer’s instructions. Specific primers for tested genes were designed on the basis of cDNA sequences from NCBI database by Primer Premier 6 software. All primers used in quantitative PCR (qPCR) are shown in Table S1. qPCR was performed on a real-time PCR machine (Bio-Rad, Hercules, CA, USA) with *TransStart* Tip Green qPCR Supermix (TRANSGEN BIOTECH, Beijing, China). The amplification reaction conditions were as follows: 95 °C for 3 min; followed by 40 cycles of 95 °C for 10 s, 60 °C for 30 s, and 95 °C for 10 s; and then a melting curve was constructed from 65 to 95 °C. The relative expression of each gene was normalized to the expression of *RPL13a* and *UBC* using the 2^−ΔΔCT^ method. Results are expressed as means ± standard deviations. Three biological replicates were used in the experiment.

### Data analysis

The raw MS/MS spectra data were searched and identified using Mascot 2.2 and Proteome Discoverer 1.4 (Thermo Fisher Scientific, Waltham, MA, USA). The database used in this study was NCBI_Nasonia_vitripennis_25492_20141104.fasta, loaded by Uniprot in November 2014. Assembled protein identifications were qualitatively analyzed using Proteome Discoverer 1.4 software. All data were reported based on 99% confidence for protein identification, as determined by a false discovery rate (FDR) of less than or equal to 1%. Statistical analysis was conducted using one-way analysis of variance (ANOVA). Results with *P* values of less than or equal to 0.05 by Tukey’s test were considered significant. Among the statistically significant proteins detected by ANOVA (*P* < 0.05), proteins showing more than 1.2-fold changes in abundance were regarded as significantly differentially expressed.

### Bioinformatics

All proteins that were found in abundance among *Wolbachia-*infected and *Wolbachia-*free group were further analyzed for functional and biological relevance. These proteins were classified by their gene functions and biological pathways using the freely available gene ontology (GO) database provided by the GO consortium. The retrieved sequences were locally searched against NCBI nr using the NCBI BLAST+ client software (ncbi-blast-2.2.28+−win32.ext) to find homologous proteins, from which functional annotations were transferred to the target proteins. The top 10 BLAST hits with an *E*-value of less than 1e^−3^ for each query protein were retrieved and loaded into Blast2GO (Version 2.7.2) for GO mapping and annotation. In addition, differentially expressed proteins, proteins participating in the immune system, and proteins involved in the reproductive process were evaluated using the Search Tool for the Retrieval of Interacting Genes/Proteins (STRING; http://string.embl.de/) to build a functional protein association network.

## Results

### Percent females of crosses and wasp longevity with different infection statuses

**Figure 1:**
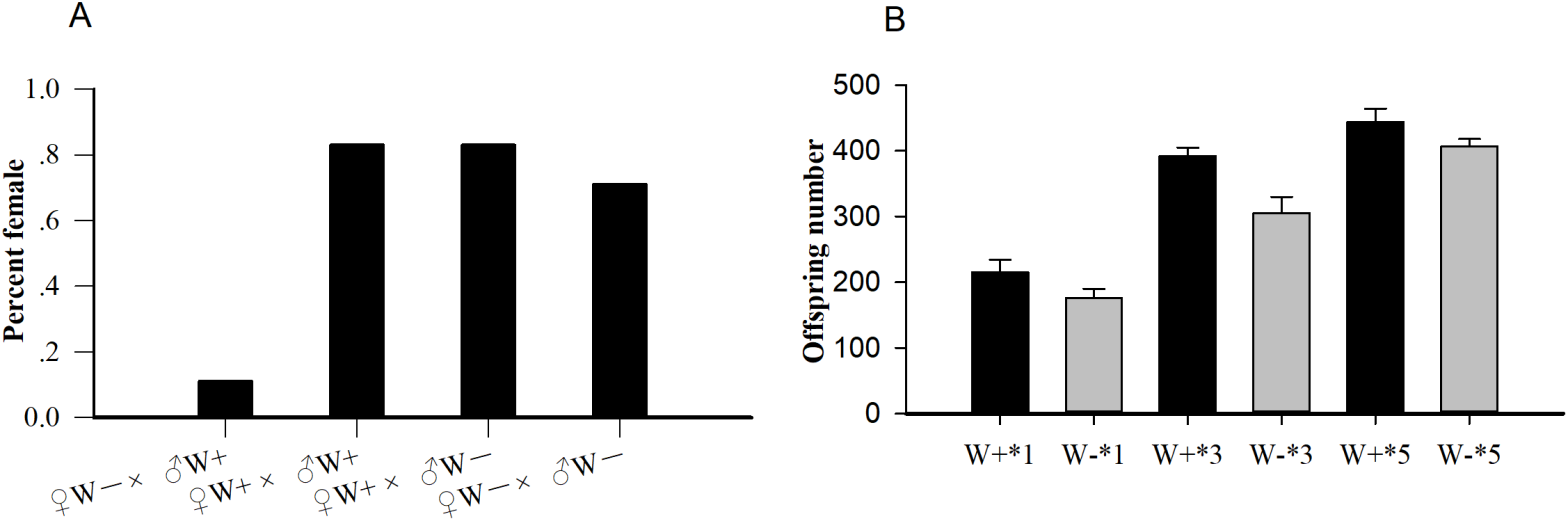
The statistics of percent females and progeny size. (A) Percent females of four cross types (♀ W− × ♂ W+, ♀ W+ × ♂ W+, ♀ W+ × ♂ W−, ♀ W− × ♂ W−) based on different *Wolbachia* infection statuses. (B) Comparisons of progeny sizes with different *Wolbachia* infection statuses and pupae numbers. W+, *Wolbachia*-infected group; W−, *Wolbachia*-free group. ♀, female, and ♂, male. The number after “*” is the number of fly pupae replaced every day.

Among the four crosses (♀ W+ × ♂ W+, ♀ W+ × ♂ W−, ♀ W− × ♂ W−, and ♀ W− × ♂ W+), the percent females of uninfected females and infected males (♀ W− × ♂ W+, 0.11) was significantly lower than that of the other three hybridization groups (*P* < 0.001; Fig. 1A), confirming that the reproductive regulation of *Wolbachia* on *N. vitripennis* was CI. The average offspring numbers of *Wolbachia*-infected wasps with one (W+*1), three (W+*3), and five pupa (W+*5) per day were 215.17 ± 19.30, 391.83 ± 12.95, and 444.17 ± 20.32 respectively, whereas the offspring numbers of *Wolbachia*-free wasps with one (W−*1), three (W−*3), and five pupa (W−*5) per day were 176.17 ± 13.53, 304.83 ± 24.86, and 407.17 ± 11.18, respectively (Fig. 1B). With the increase in pupae number, the progeny size of *Wolbachia*-infected *N. vitripennis* increased faster, implying that *Wolbachia* significantly enhanced host fecundity (one pupae: d*f* = 1, *F* = 1.572, *P* = 0.002; three pupa: d*f* = 1, *F* = 1.208, *P* < 0.001; five pupa: d*f* = 1, *F* = 2.965, *P* = 0.003).

**Figure 2:**
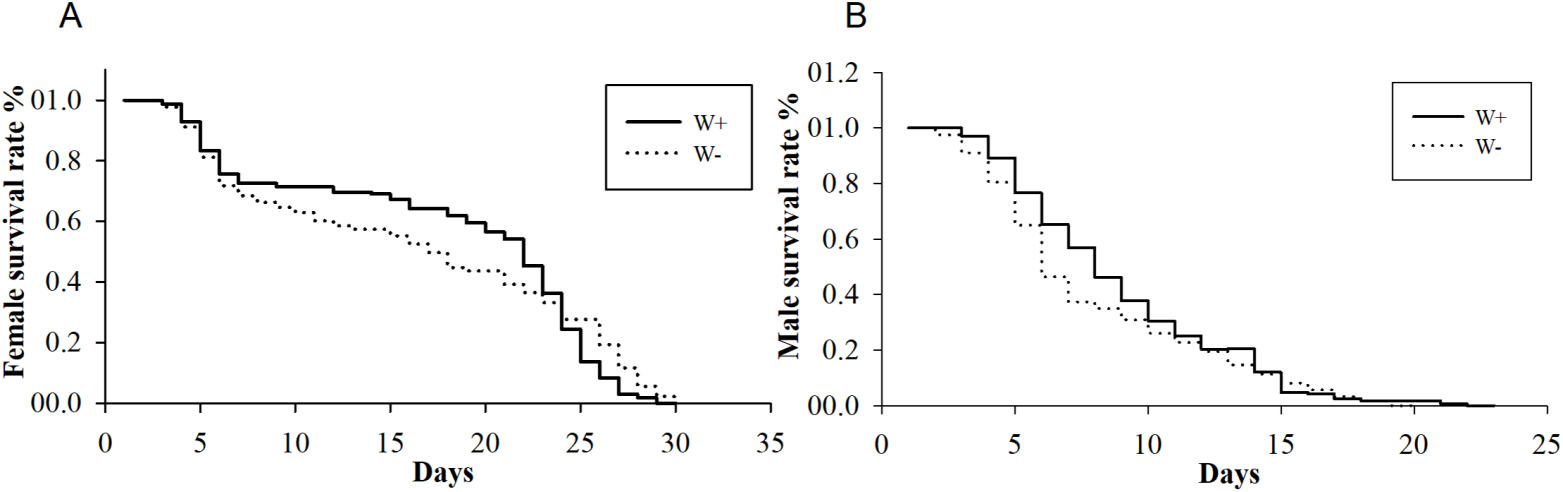
Longevity of female and male *N. vitripennis* with different *Wolbachia* infection statuses. (A) Female longevity. (B) Male longevity. “——” indicates *Wolbachia*-infected *N. vitripennis* longevity, and “-–-” indicates *Wolbachia*-free *N. vitripennis* longevity.

The average longevities of *Wolbachia-*infected and *Wolbachia-*free females were 18 and 16 days (*F*_1,288_ = 0.19, *P* = 0.07), respectively, whereas those of *Wolbachia-*infected and *Wolbachia-*free males were nine and eight days (*F*_1,347_ = 0.36, *P* = 0.12), respectively (Fig. 2). The existence of *Wolbachia* did not significantly alter the life span of *N. vitripennis*.

### Identification, GO analysis, and KEGG analysis of *Nasonia* proteomics by iTRAQ

A total of 3,109 proteins were identified from 22,946 unique peptides based on the *N. vitripennis* database with a peptide FDR of less than or equal to 0.01. From these, 3,096 proteins were quantified in three *Wolbachia*-infected and three *Wolbachia*-free *N. vitripennis* libraries (Table S2). Statistical analysis showed that 40.11% of the identified proteins had coverage greater than 20, whereas only 4.54% of proteins had coverage below 2. In addition, 2,520 proteins (81.03%) were identified with at least two peptides. Proteins with masses of more than 10 kDa had broader coverage in protein mass distribution (Fig. S1).

**Figure 3:**
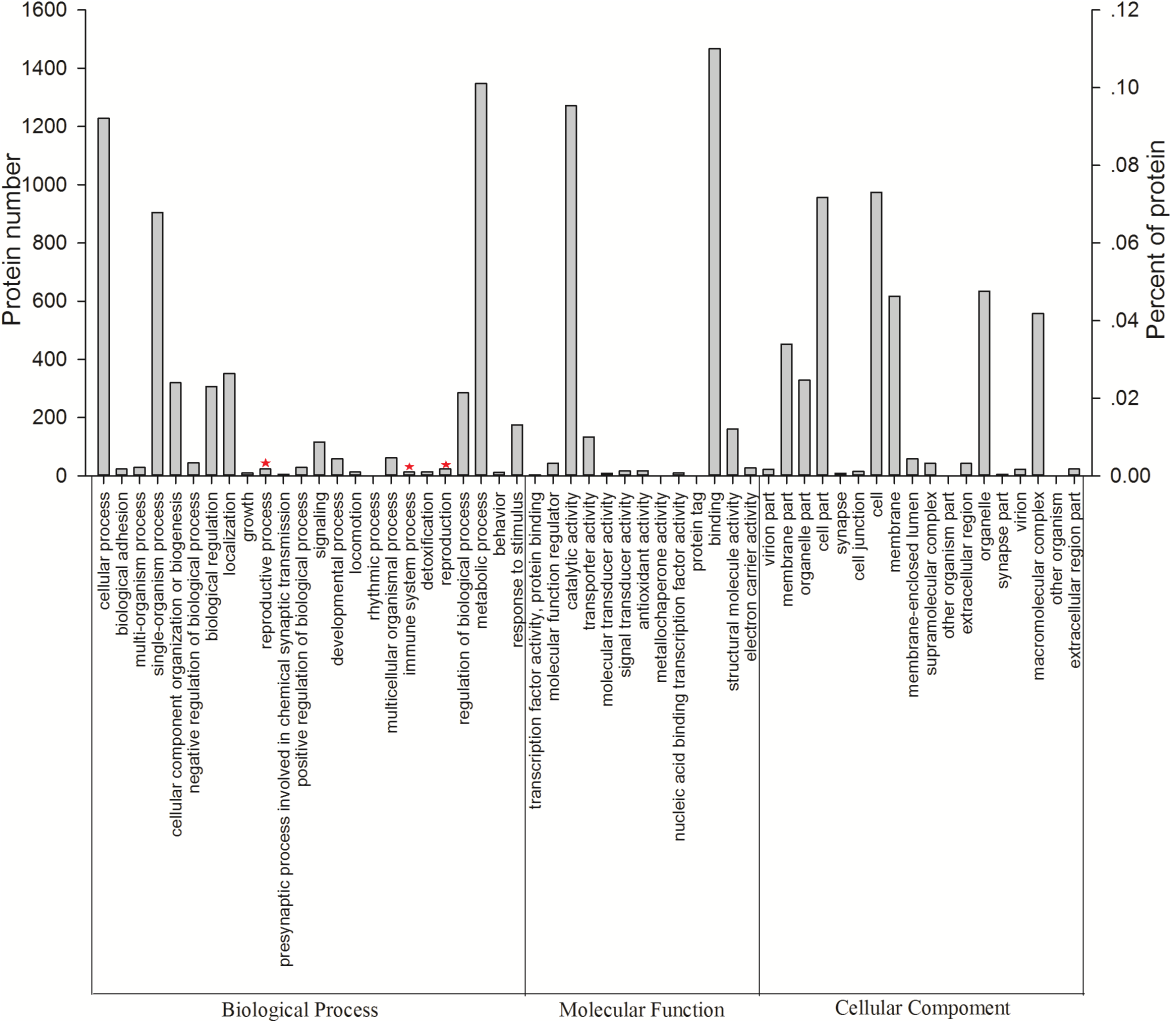
Vertical graph of gene ontology classifications for proteins identified from *Wolbachia*-infected and *Wolbachia*-free *N. vitripennis* proteomics. The proteins were found to be associated with a wide range of biological process (BP), molecular function (MF), and cellular component (CC). Immune system processes, reproductive processes, and reproduction are indicated by red stars.

All 3,096 proteins were annotated using Blast2GO (Table S3) and analyzed using the Kyoto Encyclopedia of Genes and Genomes (KEGG) pathway database (Table S4). In total, 2,578 proteins were annotated by 9,297 GO terms, covering a wide range of BPs (33.47%), MFs (41.47%), and CCs (25.26%; Fig. 3). Notably, 13 proteins participated in the immune system process, and 15 proteins were involved in both the reproductive process and reproduction (Table 1). In particular, these immune proteins were involved in regulation of the immune system process, immune response, innate immune response, and immune response-regulating cell surface receptor signaling pathways. Reproduction proteins participated in the regulation of reproductive processes, sexual reproduction, developmental processes involved in reproduction, reproductive system development, and other pathways.

**Table 1:** Immune-related and reproductive proteins based on GO functional annotations.

Accession	NV database	Description	Protein functions	W+/W−
156540814	NV18390	ADP-ribosylation factor 1	Immune	0.9413223
156547980	NV13565	Peptidoglycan recognition protein 1-like isoform X2	Immune	1.14620672
156551611	NV15422	Histone H4-like	Immune	0.96703548
283436140	NV17164	Peptidoglycan-recognition protein S2-like protein precursor	Immune	0.99915634
345486704	NV50172	Histone H3	Immune	0.93876689
345491753	NV10830	Interleukin enhancer-binding factor 2 isoform X2	Immune	0.98109544
645034592	NV13687	Guanine nucleotide-binding protein G(q) subunit alpha isoform X4	Immune	0.93153917
645037639	——	Flotillin-2	Immune	0.98390399
645038121	NV18120	Microtubule-associated protein RP/EB family member 1 isoform X1	Immune	1.01723944
156537767	NV10430	Ras-related protein Rac1, partial	Immune/reproduction	0.97298107
645015241	NV15633	Ras-related protein Ral-a isoform X5	Immune/reproduction	0.97905013
645034685	NV13706	Filamin-A isoform X1	Immune/reproduction	1.0087272
645035835	——	Superoxide dismutase [Cu–Zn]-like isoform X2	Immune/reproduction	0.92936684
156550189	NV14758	Ubiquitin-conjugating enzyme E2-17 kDa isoform X2	Reproduction	1.00353964
254910945	NV16613	Actin related protein 1	Reproduction	0.96004749
299782477	NV13256	Dynein light chain A	Reproduction	0.98722231
345494735	NV16743	Ras-related protein Rab6 isoform X2	Reproduction	0.97798355
644992919	NV14154	Histone H3.3	Reproduction	0.91800771
645001627	NV11130	Nuclear factor of activated T-cells 5	Reproduction	1.03309889
645005129	NV13395	Serine/threonine-protein phosphatase 2B catalytic subunit alpha isoform-like isoform X2	Reproduction	0.9490586
645012252	NV14708	Heterogeneous nuclear ribonucleoprotein R-like	Reproduction	1.00903299
645031721	NV12583	Tropomyosin 1 isoform X14	Reproduction	0.97277993
645034137	NV13603	Calmodulin isoform X2	Reproduction	0.97721229
645041050	NV18956	Moesin/ezrin/radixin homolog 1-like isoform X6	Reproduction	0.97626768

**Notes:**

Protein functions were determined based on GO annotations. W+/W−, the protein expression ratio of *Wolbachia*-infected and *Wolbachia*-free *N. vitripennis*. “——” indicates no corresponding proteins in the *N. vitripennis* (NV) database.

KEGG analysis showed that 1,975 proteins were assigned to 1,962 KEGG orthologies and were involved in 337 maps. The top 20 maps were analyzed (Fig. S2), and the ribosome contained the most proteins.

### Significantly differentially expressed proteins

Based on the iTRAQ-LC-MS/MS proteomic analysis, proteins with more than 1.2-fold differences and *P* values of less than 0.05 were regarded as significantly differentially expressed. There were only 23 proteins that were significantly differentially expressed between *Wolbachia*-infected and uninfected *N. vitripennis* based on the proteomics analysis, including 15 upregulated and eight downregulated proteins (Table 2). Fifteen proteins among the 23 proteins were involved in catalytic activity and binding in the MF category and in metabolic processes in the BP category. These differentially expressed proteins belonged to the biosynthesis, metabolism, and degradation processes, including Ras signaling, peroxisomes, vascular smooth muscle contraction, inflammatory mediator regulation of TRP channels, and chemical carcinogenesis. Interestingly, neither immune-related proteins nor reproductive proteins were significantly differentially expressed. Some immune genes, particularly those involved in the Toll or IMD pathways, as well as some antimicrobial peptides, were not found in either *Wolbachia*-infected or *Wolbachia*-free organisms. Protein LOC 100121395 (NV15328), which was uncharacterized in the research database, was found as putative odorant binding protein 69. Uncharacterized protein LOC1003315494 and LOC100678792 may be venom proteins by BLAST. NV11606, which was described as hydroxyacid oxidase 1, may participate not only in the carbon metabolism, but also in redox signaling and lipid homeostasis. NV16308, alcohol dehydrogenase class-3, also participated in multiple metabolic pathways, including glycolysis/gluconeogenesis, carbon metabolism, metabolism of xenobiotics by cytochrome P450, retinol metabolism, tyrosine metabolism, and fatty acid degradation.

**Table 2:** Differentially expressed proteins identified by iTRAQ between *Wolbachia*-infected and *Wolbachia*-free *N. vitripennis*.

Accession	NV database	Description	W+/W−
645042578	NV19267	Chitotriosidase-1-like, partial	0.6936338
345492571	——	Uncharacterized protein LOC100679225	0.7228549
156546540	NV12994	Cytochrome b5-like	0.8075551
156544032	NV11606	Hydroxyacid oxidase 1	0.8137581
645035666	NV12106	Acyl-coenzyme A thioesterase 13	0.825083
645005765	NV14562	Alcohol dehydrogenase-like	0.8277345
156551475	NV15328	Uncharacterized protein LOC100121395	0.8285397
645026174	NV18416	Protein henna isoform X2	0.8316617
156544652	NV11923	85/88 kDa calcium-independent phospholipase A2-like	1.2050817
345488667	SP6	Trypsin-1-like	1.2074925
345487828	——	Polyamine-modulated factor 1-binding protein 1-like	1.2602131
645009869	NV11878	Uncharacterized protein LOC100119601	1.2892213
289177071	NV17138	Carboxylesterase clade A, member 4	1.3667084
239048037	——	Venom protein V precursor	1.386467
156540800	SP31	Trypsin beta	1.390753
239735550	CCE-B2	Carboxylesterase clade B, member 2 precursor	1.3934924
238859625	NV18383	Serine protease 33 precursor	1.4075765
645027109	NV16308	Alcohol dehydrogenase class-3	1.4157821
156552724	NV18294	Endothelin-converting enzyme 1-like	1.4191134
645038835	NV12901-PA	Glutamic acid-rich protein-like	1.4199756
644992473	——	Uncharacterized protein LOC103315494	2.0601506
345485039	NV21224-PA	Uncharacterized protein LOC100678792	2.1550859
156554004	NV15950	Uncharacterized protein LOC100119759	4.0840236

**Notes:**

W+/W−, ratio of protein expression for *Wolbachia*-infected *N. vitripennis* relative to that of *Wolbachi*-free *N. vitripennis*. Downregulated proteins had a W+/W− ratio of less than or equal to 0.83, whereas upregulated proteins had a W+/W− ratio of greater than or equal to 1.2. “——” indicates no corresponding proteins in the *N. vitripennis* (NV) database.

### Quantitative real-time reverse transcription PCR

**Figure 4:**
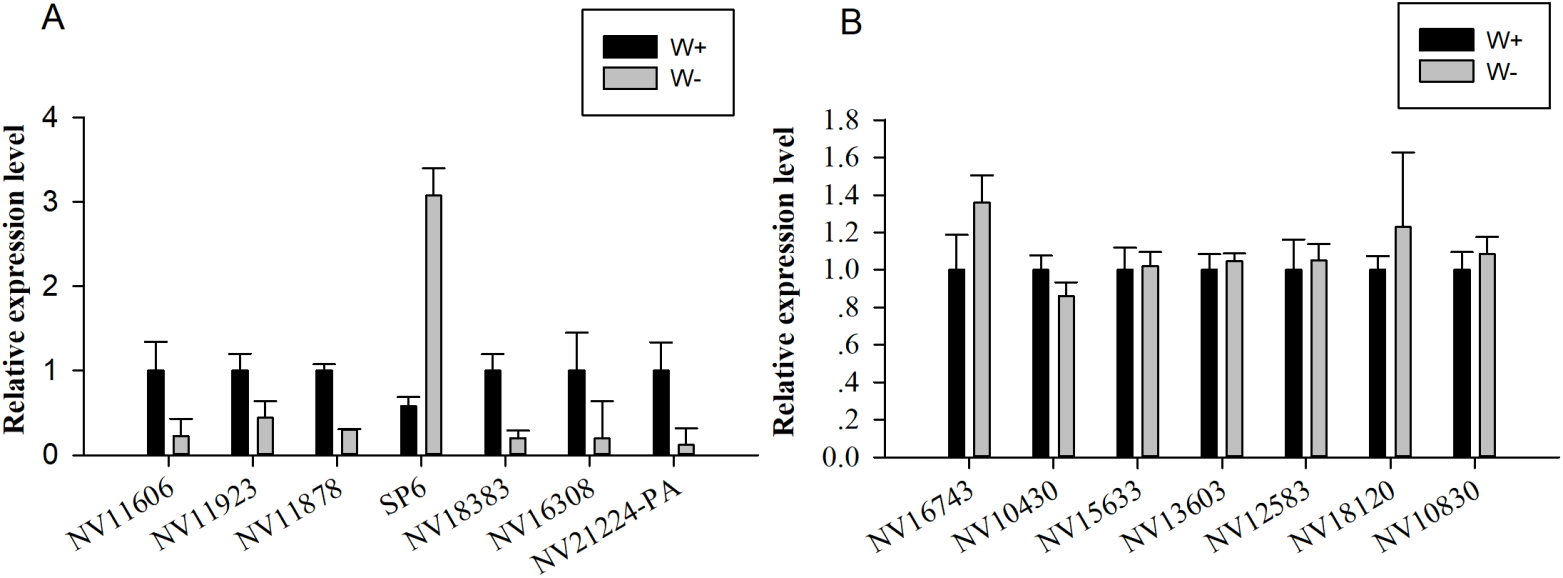
Quantitative real-time reverse transcription PCR was used for validation of selected genes. (A) Relative expression levels of genes encoding differentially expressed proteins (*NV11606*, *NV11923*, *NV11878*, *SP6*, *NV18383*, *NV16308*, and *NV21224-PA*). (B) Relative expression levels of genes encoding immune-related or reproductive proteins (*NV16743*, *NV10430*, *NV15633*, *NV13603*, *NV12583*, *NV18120*, and *NV10830*).

Seven genes encoding significantly differentially expressed proteins (*NV11606*, *NV11923*, *NV11878*, *SP6*, *NV18383*, *NV16308*, and *NV21224-PA*; Table 2) were selected for quantitative real-time reverse transcription PCR (RT-qPCR) validation; these genes encoded calcium-independent phospholipase, serine protease, alcohol dehydrogenase, hydroxyacid oxidase, trypsin, and two uncharacterized proteins. Moreover, seven genes encoding putative immune-related proteins or reproductive proteins (*NV16743*, *NV10430*, *NV15633*, *NV13603*, *NV12583*, *NV18120*, and *NV10830*; Table 1) were chosen for RT-qPCR. With the exception of *NV11606* and *SP6*, all RT-qPCR results showed similar changes compared with the proteomic analyses (Fig. 4), confirming the reliability of our proteomic analysis.

### Interactions between putative immune-related proteins, reproductive proteins, and significantly differentially expressed proteins

The online database STRING 10.0 (Search Tool for Retrieval of Interacting Genes/Proteins database) is a system that searches for interactions between known and predicted proteins based on both direct physical and functional correlations among proteins. To explore whether the significantly differentially expressed proteins influenced the immune or reproductive processes in *N. vitripennis* indirectly, the relationships among 23 significantly differentially expressed proteins, 13 proteins with putative roles in the immune system process, and 15 proteins involved in the reproductive process were studied.

**Figure 5:**
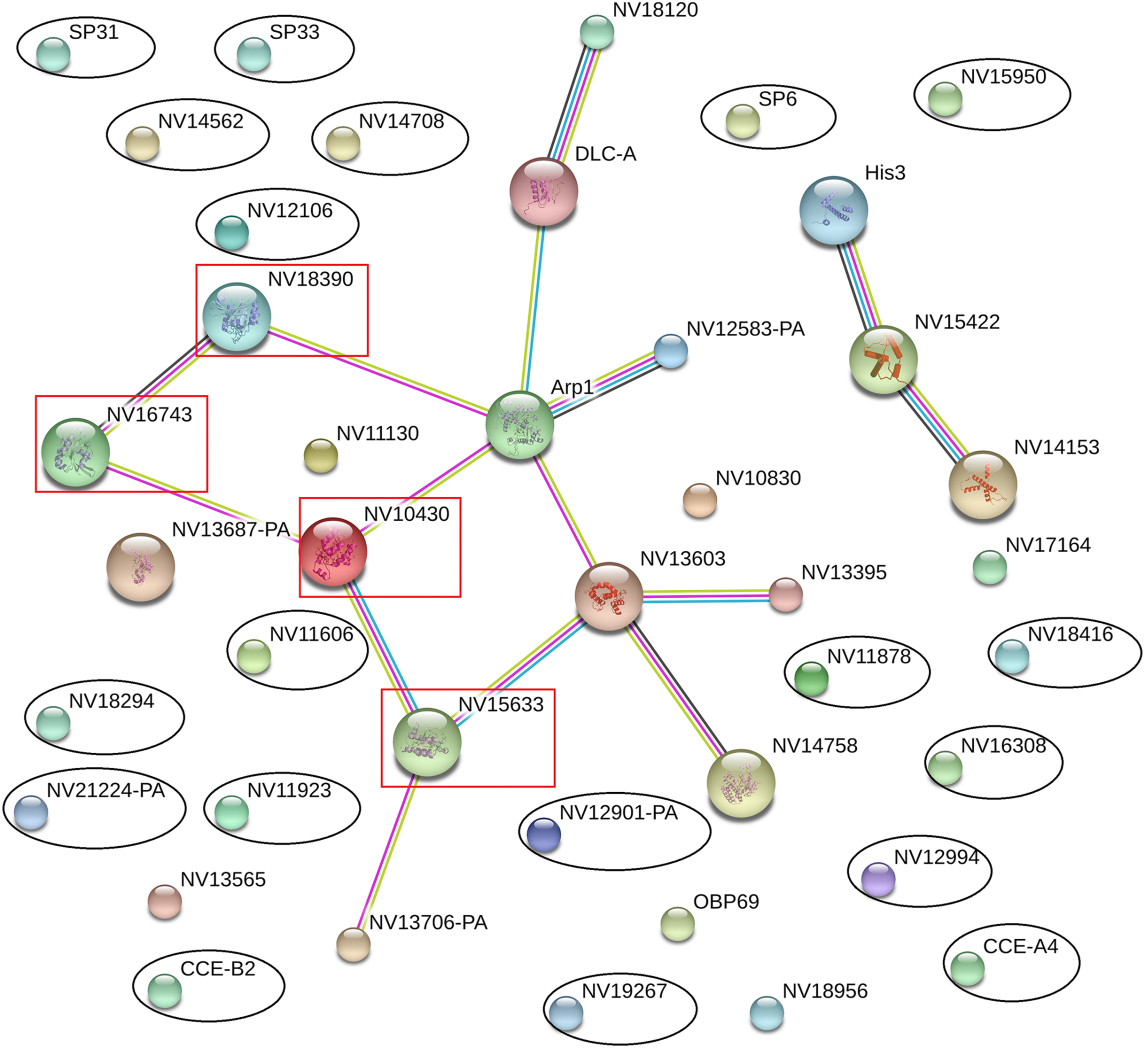
Interaction network among differentially expressed proteins, immune-related proteins, and reproductive proteins, as revealed by the STRING database. Proteins with black ovals are differentially expressed proteins, whereas proteins with red squares are Ras family proteins.

The results showed that all significantly differentially expressed proteins located on the edge of the network map were not associated with any immune-related proteins or reproductive proteins, and there were no direct interactions between significantly differentially expressed proteins (Fig. 5). Some immune-related proteins and reproductive proteins formed a network with complex interactions. For example, four Ras family proteins formed a complex interaction network, with functions in the cell cycle, cell adhesion force, cell phagocytic activity, and other processes.

### Lipid metabolic pathway

Next, we analyzed the pathways involved for all proteins from *Drosophila melanogaster*, *Aedes aegypti*, *Solenopsis invicta*, *Apis mellifera*, and *N. vitripennis* using the KEGG database and filtered out fatty acid metabolism pathways (Table 3). Fatty acid metabolism involves a total of 16 pathways. The proteins involved in the glycerophospholipid metabolism pathway, glycerolipid metabolism, and fatty acid degradation were relatively highly expressed. In *N. vitripennis*, proteins involved in primary bile acid biosynthesis were more than *D. melanogaster*, *Aedes aegypti*, *Solenopsis invicta* and *Apis mellifera*, approximately 6–13-fold.

**Table 3:** Comparison of lipid metabolism protein numbers from *D. melanogaster*, *Apis mellifera*, *Solenopsis invicta*, *Aedes aegypti*, and *N. vitripennis*.

Metabolic pathway name	*Nasonia vitripennis* (%)	*Drosophila melanogaster*	*Apis mellifera* (%)	*Solenopsis invicta* (%)	*Aedes aegypti*
00061 Fatty acid biosynthesis	12(3.34)	20(3.57)	8(2.89)	45(15.85)	13(2.91)
00062 Fatty acid elongation	12(3.34)	22(3.92)	17(6.14)	11(3.87)	29(6.50)
00071 Fatty acid degradation	26(7.24)	47(8.38)	24(8.66)	24(8.45)	41(9.19)
00072 Synthesis and degradation of ketone bodies	5(1.39)	6(1.07)	5(1.81)	6(2.11)	5(1.12)
00073 Cutin, suberine and wax biosynthesis	23(6.41)	23(4.10)	11(3.97)	21(7.39)	31(6.95)
00100 Steroid biosynthesis	22(6.13)	18(3.21)	4(1.44)	13(4.58)	30(6.73)
00120 Primary bile acid biosynthesis	33(9.19)	5(0.89)	4(1.44)	3(1.06)	3(0.67)
00140 Steroid hormone biosynthesis	23(6.41)	37(6.60)	13(4.69)	16(5.63)	41(9.19)
00561 Glycerolipid metabolism	38(10.58)	97(17.29)	38(13.72)	28(9.86)	51(11.43)
00564 Glycerophospholipid metabolism	51(14.21)	139(24.78)	59(21.30)	43(15.14)	68(15.25)
00565 Ether lipid metabolism	18(5.01)	32(5.70)	17(6.14)	9(3.17)	23(5.16)
00600 Sphingolipid metabolism	24(6.69)	41(7.31)	23(8.30)	19(6.69)	40(8.97)
00590 Arachidonic acid metabolism	28(7.80)	28(4.99)	16(5.78)	13(4.58)	24(5.38)
00591 Linoleic acid metabolism	10(2.79)	9(1.60)	8(2.89)	6(2.11)	9(2.02)
00592 alpha-Linolenic acid metabolism	12(3.34)	16(2.85)	10(3.61)	9(3.17)	13(2.91)
01040 Biosynthesis of unsaturated fatty acids	22(6.13)	21(3.74)	20(7.22)	18(6.34)	25(5.61)

**Notes:**

The metabolic pathways were analyzed using the KEGG pathway database. The entire proteomes of *D. melanogaster*, *Solenopsis invicta*, and *Aedes aegypti* were downloaded from UniProtKB, and proteomes of *N. vitripennis* and *Apis mellifera* were downloaded from the Hymenoptera Genome Database. The number and the percentage represent the protein number and proportion of the proteins involved in lipid metabolism pathway.

## Discussion

In this study, we used a combination of ecological experiments, quantitative proteomics, and bioinformatics data mining to explore the effects of native *Wolbachia* infection on *N. vitripennis*. Ecological experiments confirmed the effects of native *Wolbachia* on the reproduction of *N. vitripennis* via CI and showed that *Wolbachia* enhanced the fecundity of infected females. However, there were no significant differences in life span between *Wolbchia*-infected and *Wolbachia-*free *N. vitripennis*. In total, 3,096 proteins from *N. vitripennis* were quantified by iTRAQ; these proteins were involved in various processes, including binding activity, metabolic processes, cellular processes, and other life processes. Interestingly, the proteomes of native *Wolbachia*-infected and *Wolbachia*-free *N. vitripennis* showed few expression differences in reproduction and immune processes.

Differently expressed proteins involved in some metabolic pathways, for example, alpha-Linolenic acid metabolism, ether lipid metabolism, and carbon metabolism. It should be noted that NV18383 was known as SP33, belonging to serine protease with SP6 and SP31 (Table 2). Serine protease is a type of venom proteins, the venom glands of parasitoid wasps build an environment conducive to survival and development by influencing the arthropod host’s physiology (de Graaf et al., 2010). SP6 and SP31 were trypsin, which yielded the enzymes responsible for vital processes such as digestion, blood coagulation, fibrinolysis, development, fertilization, apoptosis, and immunity (Page & Di Cera, 2008). NV12994 was known as cytochrome *b*_5_-like, and Takamiya et al. (2016) confirmed the hypothesis that cytochrome *b*_5_ played vital roles in parasitic adaptation, together with oxygen-avid haemoglobin. Using BLAST, we found uncharacterized protein LOC1003315494 and LOC100678792 may be venom proteins as the homologous genes code venom proteins in *Trichomalopsis sarcophagae* (Martinson et al., 2017).

The immune system of insects is sensitive to the presence of symbionts (Gross et al., 2009) and plays vital roles in maintaining the host balance. Innate immunity has been extensively studied in *D. melanogaster* using expression-based methodologies, such as RNA-sequencing. One of the important biological consequences of pathogenic infection is the rapid induction of several classes of effector proteins, along with upregulation of a number of other pathway components (Apidianakis et al., 2005; De Gregorio et al., 2001, 2002; Pal, Wu & Wu, 2008; Vodovar et al., 2005). Analysis of RNA transcripts in *Nasonia* showed that infection of *Serratia marsecens* and *Enterococcus faecalis* induced upregulation of 5.6% of immune genes, compared with 1.2% of nonimmune genes, and these effects were particularly obvious for some antimicrobial peptides and recognition genes (Sackton, Werren & Clark, 2013). The homology-based *Nasonia* immune catalogue was updated by characterization of many new immune-inducible genes, some of which were taxonomically restricted to either the wasp lineage or to Hymenoptera as a whole (Sackton, Werren & Clark, 2013). In contrast, our proteomics analysis showed that natural infection by *Wolbachia* did not induce significant up- or downregulation of any immune-related proteins. We only found 13 proteins with immune-related functions in our data. Compared with the transcriptome of the homology-based *Nasonia* immune catalogue, three immune-related proteins (encoded by *NV10430*, *NV14758*, and *NV17164*) were found in the catalogue, but were not significantly regulated by short-term infection with *Serratia marsecens* or *E. faecalis*.

Transfection of *Wolbachia* has caused concerns in mosquitos because this endosymbiont may be used for control of Dengue virus (Ruang-Areerate & Kittayapong, 2006; Schnettler et al., 2016; Walker et al., 2011; Zhang, Hussain & Asgari, 2014). Short-term infection with *Wolbachia* reduces reproductive regulation in insect hosts, such as CI, male-killing, and sperm modification (Blagrove et al., 2013; Jeffries & Walker, 2016; McGraw et al., 2001). Studies have shown that short-term infection with *Wolbachia* significantly affects the expression of immune genes, including those involved in the Toll and IMD immune pathways, as well as a number of antimicrobial genes (Bian et al., 2013; Pan et al., 2012; Rances et al., 2012). In contrast, in long-term native *Wolbachia* infection, induction of Toll-like receptors or IMD immune-related genes is not observed in *Aedes fluviatilis* mosquitos (Caragata et al., 2017). Our proteomics analysis of *N. vitripennis* also showed that no immune proteins were significantly differentially expressed following *Wolbachia* infection. We speculate that insect hosts may have adapted to infection by endosymbiotic *Wolbachia* through long-term intergrowth by adjusting its physiological and biochemical activities in vivo. As proposed by Zug, a recently acquired infection is likely to trigger immune responses as a key resistance mechanism, whereas in co-evolved associations, resistance may no longer be the best response to infection (Zug & Hammerstein, 2015b). The absence of systemic immune activation is not rare among native *Wolbachia* infections and may be symptomatic of increased tolerance on the part of the host and reduced pathogenicity on the part of the symbiont (Bourtzis, Pettigrew & O’Neill, 2000; Caragata et al., 2017). This could explain our proteomic results to some extent. Notably, stable infection with *Wolbachia* in *D. melanogaster* has been shown to yield different results. Gene expression analysis of the testes dissected from third instar larvae of both *Wolbachia-*infected and *Wolbachia*-free *D. melanogaster* showed that a total of 296 genes were significantly identified by microarray analysis. Differential expression of genes related to metabolism, immunity, reproduction, and other functions was observed (Zheng et al., 2011). Moreover, proteomics analysis showed that 83 proteins, including 14 and five proteins involved in reproduction and immunity, respectively, were significantly differentially expressed between one-day-old *Wolbachia-*infected and one-day-old *Wolbachia-*free *D. melanogaster* (Yuan et al., 2015). We used local BLASTp to search those 83 significantly differentially expressed proteins in our *N. vitripennis* proteomics data and found 10 proteins with more than 70% identity (*E* score < 10^−5^), as shown in Table S5. In particular, three homologous proteins (NCBI ID: 299782477, 156537767, 645012252) were involved in reproduction, and protein 156537767 was also involved in immunity. However, these three proteins did not show significantly differential expression in our proteomics analysis of native *Wolbachia*-infected and *Wolbachia*-free *N. vitripennis*.

As a parasitoid, *N. vitripennis* is a carnivore, feeding on an amino acid-rich diet both as larva and adults (Werren & Loehlin, 2009a). The *Nasonia* genome revealed loss or rearrangement of some amino acid metabolic pathways, including tryptophan, and aminosugar metabolism, which may reflect its specialized carnivorous diet (Werren et al., 2010). We hypothesized that *Nasonia* may have more lipid metabolism proteins; thus, we compared the numbers of lipid metabolism proteins from *D. melanogaster*, *Aedes aegypti*, *Solenopsis invicta*, *Apis mellifera*, and *N. vitripennis* (Table 3). To our surprise, with the exception of more proteins involved in primary bile acid biosynthesis, *Nasonia* did not exhibit changes in lipid metabolism genes compared with four other insect species. Our proteomics analysis of *Nasonia*, as a model insect, provided important insights that will facilitate the elucidation of various metabolic and immune-related mechanisms.

The interactions between insect hosts and symbionts are being extensively studied, particularly with regard to unknown molecular mechanisms. Our current findings provided novel insights into the complex interactions between *Nasonia* and native *Wolbachia* infection. Based on our findings, we propose the gradual adaptation of *Wolbachia* in insects, although some phenotype is obvious. With long-term coexistence, both insect hosts and symbionts may adapt to each other. More studies are needed to verify this hypothesis.

## Conclusions

In this study, we evaluated the proteomics of *N. vitripennis* and the effects of native infection by the widespread endosymbiont *Wolbachia* using iTRAQ-LC-MS/MS. In total, 3,096 proteins were identified from native *Wolbachia*-infected and antibiotic-treated *Wolbachia*-free *N. vitripennis* samples, including a wide range of proteins involved in BPs, MFs, and CCs. Interestingly, although the phenotype was obvious, there were few significant changes in immune or reproductive proteins between samples with different *Wolbachia* infection statuses, and most differentially expressed proteins belonged to categories of binding processes, catalytic activity, and metabolic processes. Furthermore, there were no direct correlations in differential expression between immune and reproductive proteins. Our findings provided valuable insights into the mechanisms underlying the interactions between insect hosts and endosymbionts.

## Supplemental Information

10.7717/peerj.4905/supp-1 Supplemental Information 1 Fig. S1. Proteins mass distribution.

10.7717/peerj.4905/supp-2 Supplemental Information 2 Fig. S2. Enriched KEGG pathway analysis of proteins quantified from *N. vitripennis* proteomics by iTRAQ. The top 20 pathways are shown.

10.7717/peerj.4905/supp-3 Supplemental Information 3 Table S1. Primers for qPCR.

10.7717/peerj.4905/supp-4 Supplemental Information 4 Table S2. Proteins information quantified by iTRAQ.

10.7717/peerj.4905/supp-5 Supplemental Information 5 Table S3. Details of gene ontology classifications for proteins identified from *Wolbachia*-infected and *Wolbachia*-free *N. vitripennis* proteomics.

10.7717/peerj.4905/supp-6 Supplemental Information 6 Table S4. KEGG pathway analysis.

10.7717/peerj.4905/supp-7 Supplemental Information 7 Table S5. Proteins with more than 70% identity analysed by local BLASTp. W+/W-, the protein abundance ratio of *Wolbachia*-infected *N. vitripennis* compared to *Wolbachia*-free *N. vitripennis*. Identities were analysed by local BLASTp (E-value < 10-5).

## Additional Information and Declarations

### Data Availability

The following information was supplied regarding data availability: The raw data has been submitted to the iProX under accession number IPX0001173001.
