# Supplementary figures and images for: Proteomics of Nasonia vitripennis and the effects of native Wolbachia infection on N. vitripennis
Source: PeerJ. 2018 May 28;6:e4905. doi: 10.7717/peerj.4905 (PMC5978391; doi:10.7717/peerj.4905)

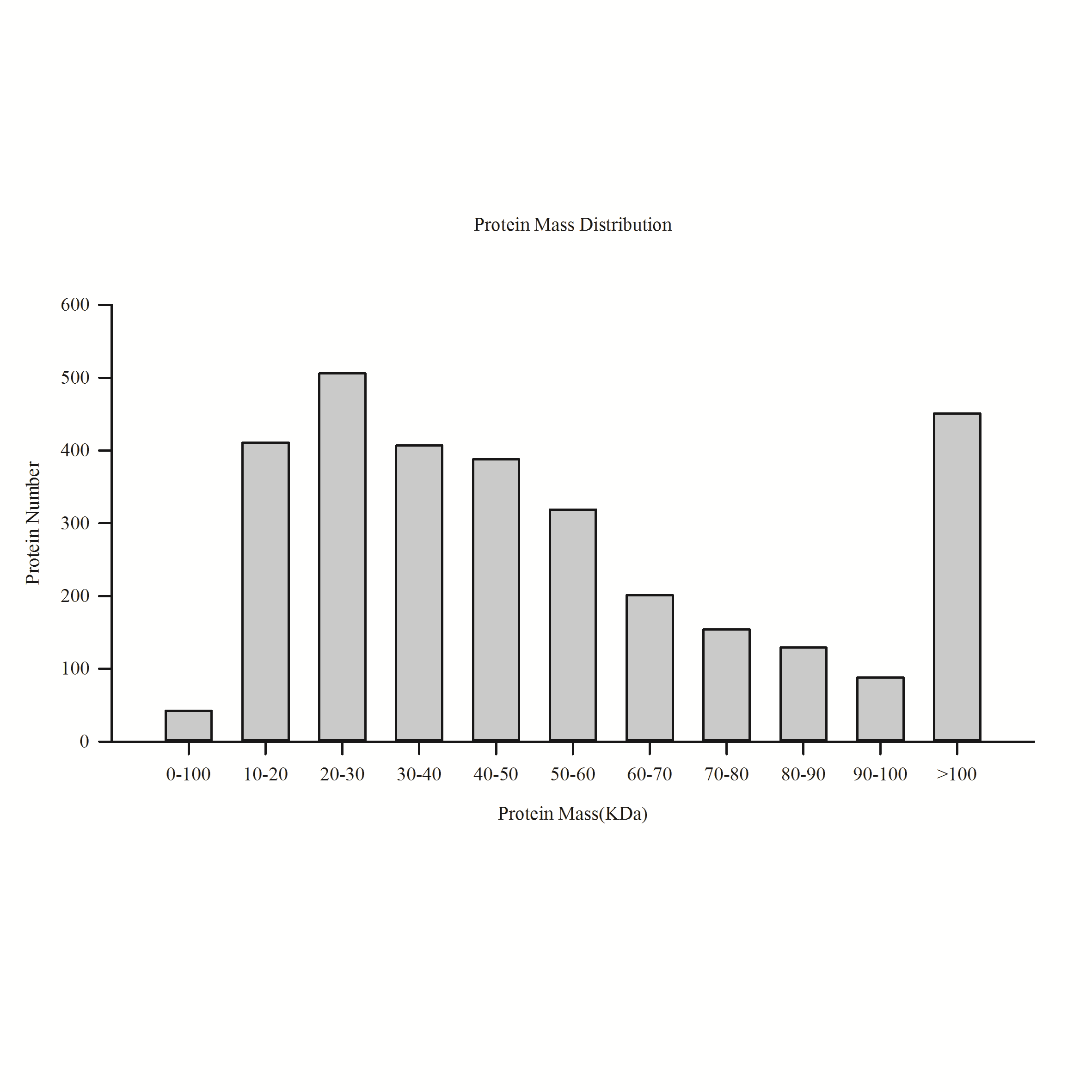

Supplement: Supplemental Information 1 [file peerj-06-4905-s001.png]

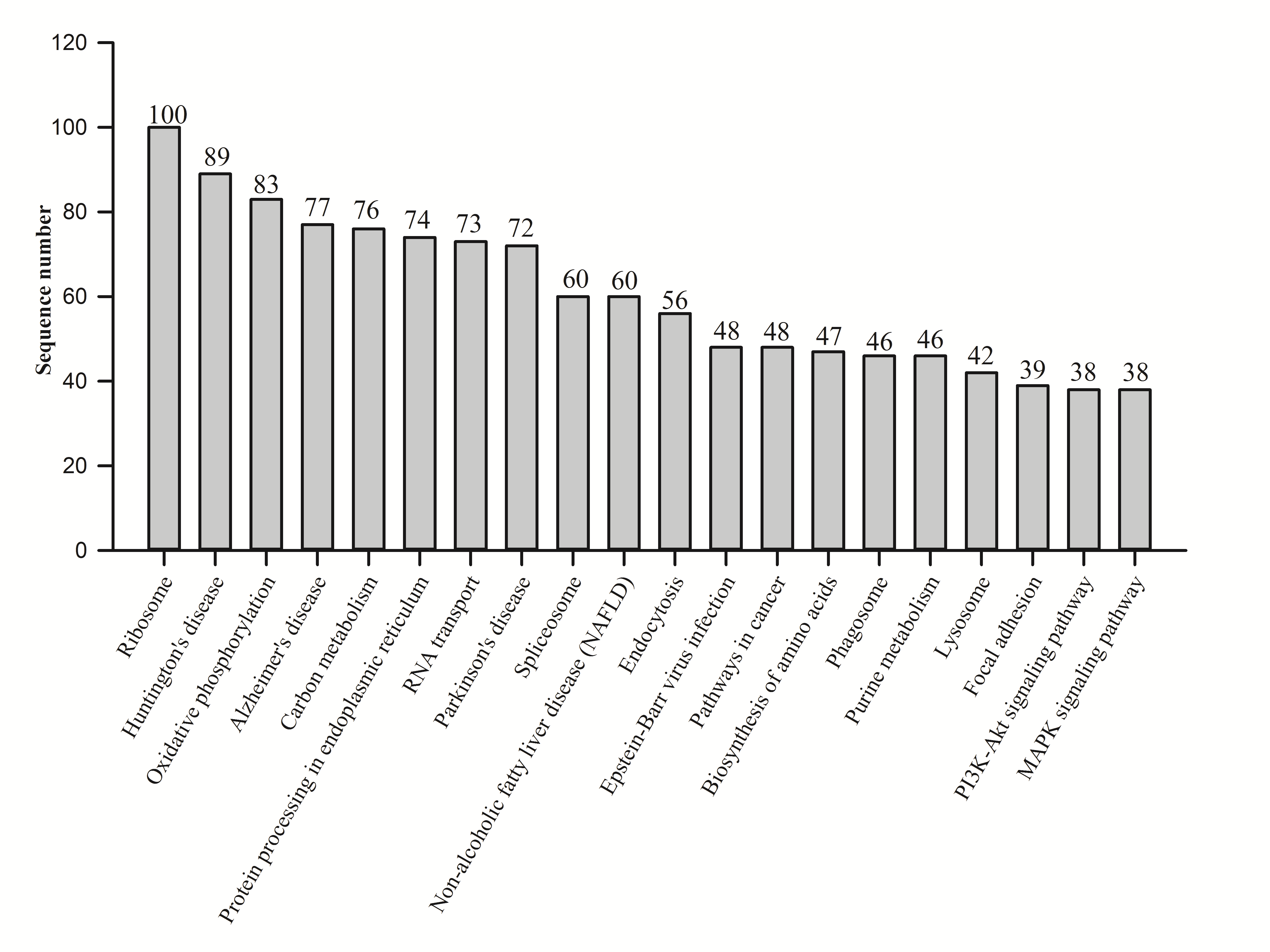

Supplement: Supplemental Information 2 — The top 20 pathways are shown. [file peerj-06-4905-s002.png]
